# Hands-On Parameter Search for Neural Simulations by a MIDI-Controller

**DOI:** 10.1371/journal.pone.0027013

**Published:** 2011-10-31

**Authors:** Hubert Eichner, Alexander Borst

**Affiliations:** Max-Planck-Institute of Neurobiology, Department of Systems and Computational Neurobiology, Martinsried, Germany; Mount Sinai School of Medicine, United States of America

## Abstract

Computational neuroscientists frequently encounter the challenge of parameter fitting – exploring a usually high dimensional variable space to find a parameter set that reproduces an experimental data set. One common approach is using automated search algorithms such as gradient descent or genetic algorithms. However, these approaches suffer several shortcomings related to their lack of *understanding* the underlying question, such as defining a suitable error function or getting stuck in local minima. Another widespread approach is manual parameter fitting using a keyboard or a mouse, evaluating different parameter sets following the users intuition. However, this process is often cumbersome and time-intensive. Here, we present a new method for manual parameter fitting. A MIDI controller provides input to the simulation software, where model parameters are then tuned according to the knob and slider positions on the device. The model is immediately updated on every parameter change, continuously plotting the latest results. Given reasonably short simulation times of less than one second, we find this method to be highly efficient in quickly determining good parameter sets. Our approach bears a close resemblance to tuning the sound of an analog synthesizer, giving the user a very good intuition of the problem at hand, such as immediate feedback if and how results are affected by specific parameter changes. In addition to be used in research, our approach should be an ideal teaching tool, allowing students to interactively explore complex models such as Hodgkin-Huxley or dynamical systems.

## Introduction

A frequent challenge in computational neuroscience is to come up with models that properly replicate some quantitative or qualitative characteristics of measured data. This process usually involves exploring a high-dimensional space of model parameters in order to arrive at satisfying fits of the data in question. One common approach is to use an automated search algorithm such as gradient descent [1], variants of Newton's method [2] or genetic algorithms [3]. While successfully applied in a large range of projects (reviewed in [4]), these methods also suffer from several shortcomings, such as requiring an exactly defined measure of quality for a given parameter set (an *error function*), slow convergence or ending up in local minima. Most notably, the search algorithm is usually unaware of the intrinsic structure of the underlying problem.

A particularly profound problem, however, becomes apparent when the user desires a good qualitative, not quantitative fit, for instance because the model is intentionally simplified and thus unable to exactly reproduce the results, or because the desired behavior of the model is hard to formulate in mathematical terms. In such cases, standard error functions like the root mean squared difference between the actual and the target output may fail to arrive at suitable parameter sets, and significant effort is spent on defining and refining a proper error function. A further problem is optimizing multiple objectives at the same time [5] when the tradeoff between the different objectives depends on the user's intuition.

Consequently, a further frequently employed approach is manual parameter fitting ([6], [7]). Here, the user evaluates different parameter sets, modifies them according to his intuition of the problem, evaluates them again etc. This process usually involves a lot of typing and mouse movements, and is often a cumbersome and tedious procedure.

Inspired by the learning process on musical instruments or synthesizers, where the constant auditory feedback shapes the student's technical abilities, we treat the process of parameter adjustment in a similar way. The parameters are now controlled by a MIDI device, and the model is continuously simulated with the actual parameters, repeatedly plotting the latest result on the screen.

## Materials and Methods

An Evolution UC-33 USB MIDI controller (M-AUDIO/Avid Technology Inc., USA) attached to a PC (Intel Core 2 Quad Q9550, 2.83 GHz, 8 GB main memory, running Windows 7) is accessed by a middleware software layer (see File S1) written in the Java programming language (Oracle Corporation, Redwood Shores, CA; compiled with JDK version 1.6). This program is notified about control element changes via the javax.sound.midi application programming interface and stores the positions of the modified control elements. The middleware is then accessed by the simulation software. We employed either the MATLAB (Mathworks, Natick, MA; JRE version 1.6) or IDL (ITTVIS, Boulder, CO; JRE version 1.6) development environment which both are unable to directly access MIDI devices by default but can readily communicate with Java software. The information about control element changes is retrieved by the simulation software, which then adjusts the model parameters, reruns the simulation and plots the most recent results on the screen. While the actual implementation of the graphical user interface is left completely to the user, we found it helpful to illustrate the control elements and their positions right next to the simulation results; this way, the user does not have to shift attention from the MIDI controller to the screen and vice versa. In its current version, the middleware software layer supports only one MIDI controller, but it could be enhanced to support multiple devices to increase the number of control elements.

While this concept has been applied to multiple problems in our lab, we here present a popular example for illustration purposes, the simulation of the Hodgkin-Huxley equations [8]. The spiking mechanism is formally described by the following current balance equation for one compartment:
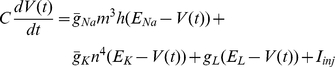
with 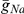 and 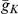 denoting the maximum sodium and potassium conductance, respectively, and with the gating variables defined by
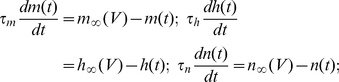
We assume voltage-independent, but user-controlled values for the time constants, and approximate the steady-state functions of the gating variables by a sigmoidal function of the form
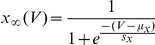
This slight deviation from the original Hodgkin-Huxley allows for easier modification of the time constant and the activation curve, the latter by shifting it on the voltage axis (μ_x_) or changing its slope (s_x_).

The sodium and potassium conductances are computed using the explicit Euler scheme; the final equation for computing the voltage at the next time step is then obtained by applying the implicit Euler method for greater numerical stability:
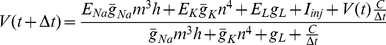
We use a time step of Δt = 0.1 ms and simulate the model for 300 ms, with a step current injection of I nA lasting from t = 50 ms to t = 250 ms. The eleven model parameters τ_m_, τ_h_, τ_n_, μ_m_, μ_h_, μ_n_, s_m_, s_h_, s_n_, 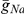, 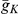 and the current amplitude I are set via the MIDI controller. The Java middleware layer is polled for control element changes in an infinite loop. Each time a change is observed, the corresponding parameters are adjusted, the model is simulated with the new parameter set, and the new result is immediately plotted on the screen.

## Results

We attached a MIDI controller featuring knobs, sliders and buttons to the computer performing the simulations. The model parameters and the simulation results are updated whenever the position of a MIDI control element is changed. This leads to a more or less instant change of the depicted results, depending on the simulation runtime. The method therefore provides a closed-loop experience to the user. Indeed, and similar to using a musical instrument or tuning the sound of an analog synthesizer, we find that being able to instantly and, equally important, continuously observe changes in the results based on manually controlling the knobs and sliders gives a certain intuition for the problem at hand. This allows for a rapid exploration of high-dimensional parameter spaces and identifying potential solutions to a certain problem. It also should make this technique a great tool for teaching.

**Figure 1:**
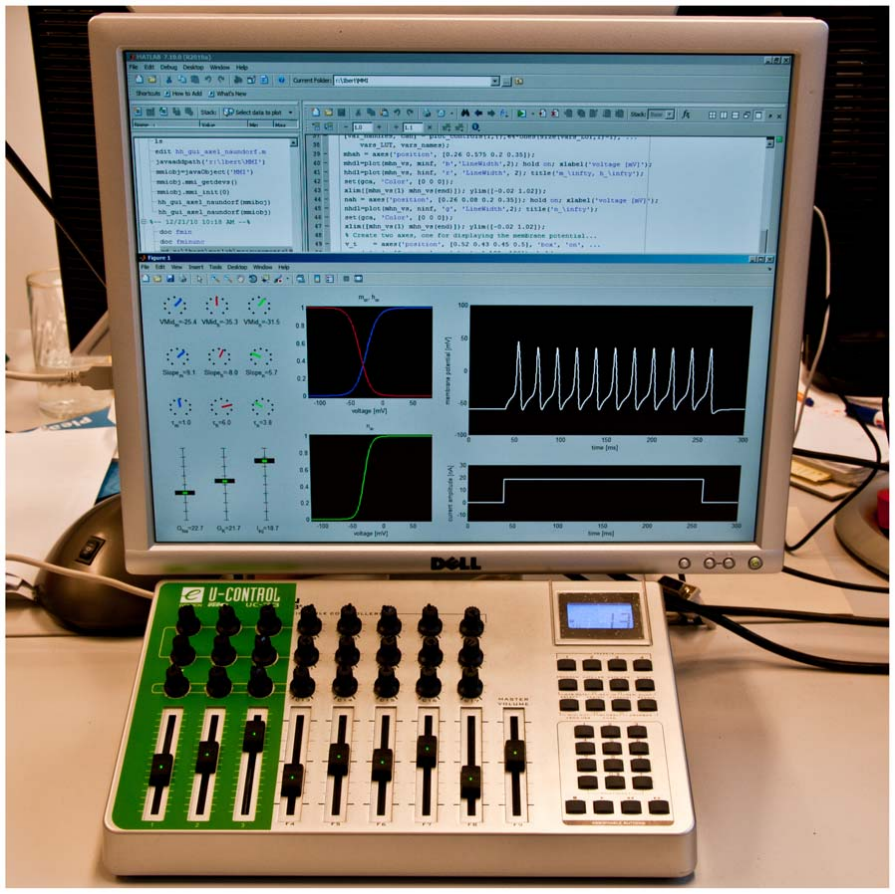
Using a MIDI device to control a computer simulation. On the computer screen, the application (in this case, a simulation of the Hodgkin-Huxley model) is depicted, with the actually used control elements of the MIDI controller (highlighted by a green template) replicated on the left side of the application. The simulation is updated on every control element change, and the latest results are immediately plotted on the right side of the application window.

To illustrate this method, we chose one of the most popular models in neuroscience, the Hodgkin-Huxley equations for an excitable patch of membrane [8], in a slightly modified form to allow for greater user control of the ion channel activation and inactivation process (see Materials & Methods). Figure 1 depicts a graphical user interface written in MATLAB. The knobs and sliders on the MIDI controller that are assigned to the various changeable parameters are replicated in the left portion of the window; this way, the user does not have to switch attention between the controller and the computer screen. Changes on a knob or slider instantly change the internal parameters of the simulation, and the most up-to-date activation curves, current injection and voltage traces are plotted with a latency of about 80 ms.

It is straightforward to use our middleware layer for parameter fitting. It is written in Java, which allows for accessing the controller from various development environments that do not natively support MIDI devices but allow accessing Java classes, such as MATLAB or IDL, but also using other programming language capable of interfacing Java. Briefly, the middleware layer has to be initialized with one command; after that, a single function returns tuples of the form (control element ID, value) when a knob, switch or button has been turned or pressed. The user has to modify existing programs only slightly to continuously check for events from the controller, followed by translating the received values into specific parameter changes. Whether the relation between the control element value and the parameter is linear, exponentially etc. is left to the user. On rare occasions where either the dynamic range or the precision of a knob or slider is found to be insufficient, we assigned other knobs that dynamically adjust the parameter range or precision of the corresponding primary control element. For instance, if a simulation parameter is obtained by linearly mapping the knob position to the interval [low; high], then a second knob might be used to change either low or high (on an exponential scale), or both simultaneously by widening or shortening the interval.

**Figure 2:**
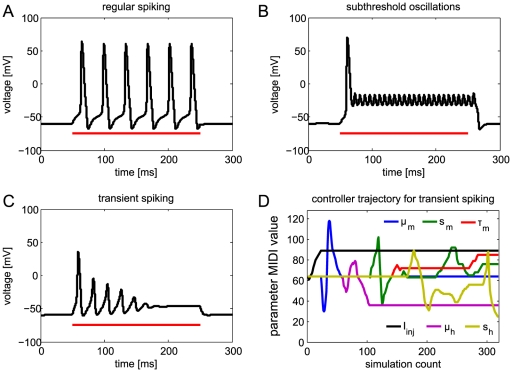
Simulation of the Hodgkin Huxley model with three example parameter sets. The red line shows the duration of the current injection. (A) Regular spiking behavior. (B) A parameter set that leads to subthreshold oscillations. (C) A parameter set that exhibits transient spiking upon current injection. (D) Trajectory of the modified parameters during fitting of the model in C. The ordinate ranges from 0 to 127, the range of control elements on a MIDI controller. The abscissa does not depict absolute time but the simulation index.

Figure 2 shows three example fits of the Hodgkin-Huxley model responding to a current injection step (red trace): regular spiking (Fig. 2A), subthreshold oscillations (Fig. 2B) and a parameter set that exhibits transient spiking (Fig. 2C). Figure 2D depicts the control element changes performed by the user for the six parameters that were varied to arrive at the results of Figure 2C. The ordinate corresponds to the value reported by the MIDI controller for a specific knob or slider, which lies between 0 and 127. It can be seen that some parameters are changed only once (current injection strength I_inj_), while others are found to be negligible (midpoint of sodium activation curve μ_m_) or changed multiple times, even at once (s_m_ and s_h_).

## Discussion

We presented a novel method for manual parameter fitting that provides the user with an external device for adjusting the model's variables. For illustrational purposes, we restricted our results to the relatively simple example of simulating the Hodgkin-Huxley equationhowever, we have used the method on multiple occasions for more complex tasks where defining an exact error function was difficult.

The major limitation of our technique is latency, that is, the delay between a user-controlled parameter change on the controller and the actual display of the simulated results based on the new parameter set. While the delay between e. g. turning a knob and changing the corresponding parameter in the simulation was found to be negligible, simulation times of about a second or more lead to problems. To draw another analogy to musical instruments, imagine trying to learn the piano when each time a key is pressed, it takes a second until the actual tone can be heard. In a similar way, runtimes in the order of seconds limit the intuition the user can gain for a model. Several classes of problems, such as complex multi-compartment simulations with active membrane parameters, or simulations of Integrate & Fire neurons that require many repetitions to form a reliable firing rate estimate, are therefore likely excluded from this technique in the near future, at least on single PCs.

We would like to point out, however, that these challenges may be alleviated by using coarser discretization steps, e. g. increasing the time step size of simulations or reducing the number of compartments. Other possibilities are careful optimization of the source code, such as computing the voltage-dependent forward and backward rates of ion channels of the Hodgkin-Huxley model in advance and using look-up tables during the simulation, as described in the previous section. In addition, parallelizing the simulation by e. g. multi-threading, as already employed in MATLAB for basic vector and matrix operations, and further advances in microprocessor development may also render the method more realistic in the future for an even broader class of problems.

Although only 12 knobs and sliders were used in the Hodgkin-Huxley example, there is no software-imposed restriction on the supported number of control elements; the MIDI controller we used has 33 knobs and sliders and, in addition, 14 buttons. It is possible to control more parameters by assigning a control element to several simulation variables and switch between these assignments by using push buttons [9]. In addition, a coherent user experience upon such switches would require a MIDI controller where the sliders are motor-controlled to move them to the correct position after an assignment switch, an option we are currently investigating. A further possibility is to connect multiple MIDI controllers, but this would require changes to the Java middleware.

Finally, we would like to stress the applicability of our method to teaching. Every senior scientist will agree that a certain familiarity with a method or a model is best achieved by virtually playing around with it and putting it to use. Accordingly, rather complex models such as non-linear dynamical systems could, in addition to the theoretical introduction, be taught by providing students with the simulation and a controller, allowing them to become familiar with the problem and solve exercises such as finding bifurcation points or optimal solutions to specific questions.

## Supporting Information

File S1 **Source code for the MIDI interface.**
(DOC)
