# Supplementary material for: Hands-On Parameter Search for Neural Simulations by a MIDI-Controller
Source: PLoS One. 2011 Oct 31;6(10):e27013. doi: 10.1371/journal.pone.0027013 (PMC3205000; doi:10.1371/journal.pone.0027013)
Supplement: File S1 — Source code for the MIDI interface. (DOC) [file pone.0027013.s001.doc]

The source code for the Hodgkin-Huxley GUI and a technical introduction of the source code are available from the authors. The source code for the MIDI interface is listed below.

/**********************************************************

* MATLAB MIDI Interface *

* Copyright 2011 Hubert Eichner *

* Max-Planck-Institute of Neurobiology *

* Department of Systems and Computational Neurobiology *

* Martinsried, Germany *

* eichner (at) neuro.mpg.de *

* This program is free software: you can redistribute it *

* and/or modify it under the terms of the Lesser GNU *

* General Public License as published by the Free *

* Software Foundation, either version 3 of the License, *

* or (at your option) any later version. *

* This program is distributed in the hope that it will be *

* useful, but WITHOUT ANY WARRANTY; without even the *

* implied warranty of MERCHANTABILITY or FITNESS FOR A *

* PARTICULAR PURPOSE. See the Lesser GNU General Public *

* License for more details, available from *

* <http://www.gnu.org/licenses>. *

**********************************************************/

import javax.sound.midi.*;

import java.util.Vector;

class MMI_Recv_Msg {

int[] data;

public MMI_Recv_Msg(int ch, int cmd, int data1, int data2) {

data = new int[4];

data[0] = ch; data[1] = cmd;

data[2] = data1; data[3] = data2;

}

int[] getData() {

return data;

}

}

class MMI_Recv implements Receiver {

private Vector<MMI_Recv_Msg> msgs;

public MMI_Recv() {

msgs = new Vector<MMI_Recv_Msg>();

}

public int[][] recv_read() {

int nmsgs = msgs.size();

int[][] ret;

MMI_Recv_Msg msg;

if (nmsgs == 0) {

return null;

}

ret = new int[nmsgs][];

for (int i=0; i<nmsgs; i++) {

ret[i] = msgs.remove(0).getData();

}

return ret;

}

public void close() {

System.out.println("closing...");

}

public void send(MidiMessage msg, long timeStamp) {

ShortMessage msg_s = (ShortMessage)msg;

msgs.add(new MMI_Recv_Msg(msg_s.getChannel(),

msg_s.getCommand(), msg_s.getData1(),

msg_s.getData2()));

}

}

public class MMI {

private MidiDevice.Info[] mdi;

MidiDevice device;

private MMI_Recv recv;

private Transmitter trans;

public void MMI() {

}

public String[] mmi_getdevs() {

String[] ret;

mdi = MidiSystem.getMidiDeviceInfo();

ret = new String[mdi.length];

for (int i=0; i<mdi.length; i++) {

ret[i] = mdi[i].getName();

}

return ret;

}

public void mmi_init(int devid) {

mdi = MidiSystem.getMidiDeviceInfo();

recv = new MMI_Recv();

try {

device = MidiSystem.getMidiDevice(mdi[devid]);

device.open();

trans = device.getTransmitter();

} catch (MidiUnavailableException e) {

System.out.println("Error initializing"+

"MIDI controller");

}

trans.setReceiver(recv);

}

public int[][] mmi_read() {

return recv.recv_read();

}

}

Copying the above source code into a file named MMI.java and compiling it with the Java compiler produces a file called MMI.class. This class file must be copied into MATLAB's java search path (see the javaaddpath command). To use the MIDI controller, enter the following commands in MATLAB:

mmiobj = javaObject('MMI');

mmiobj.mmi_getdevs(); % gives a listing of available MIDI devices

mmiobj.mmi_init(0); % init the desired MIDI device (here: #0)

mmiobj.mmi_read(); % return buffered MIDI messages containing

% control element Ids and their position

The function mmiobj.mmi_read() returns all buffered MIDI messages and is typically called from within an infinite loop. It may be necessary to use an older Java compiler (e.g. from JDK1.6) to remain compatible with MATLAB’s Java version.
